# Compliance potential mapping: a tool to assess potential contributions of walking towards physical activity guidelines

**DOI:** 10.1186/1471-2458-14-511

**Published:** 2014-05-27

**Authors:** Md Moniruzzaman, Antonio Páez, Catherine Morency

**Affiliations:** 1 School of Geography and Earth Sciences, McMaster University, 1280 Main Street West, Hamilton, ON L8S 4 K1, Canada; 2 Département des génies civil, géologique et des mines, École Polytechnique, 2900, boul. Édouard-Montpetit, Montréal, QC H3C 3A7, Canada

## Abstract

**Background:**

Walking for transport is increasingly considered an important component for meeting physical activity guidelines. This is true for individuals of all ages, and particularly important for seniors, for whom other physical activities may not be recommended. In order to evaluate the potential contributions of walking to physical activity, in this paper the concept of Compliance Potential Mapping is introduced. The concept is illustrated using seniors as a case study.

**Methods:**

Based on estimates of walking trip distance and frequency, estimates of expected total daily walking distance are obtained. These estimates are converted to weekly walking minutes, which are in turn compared to recommended physical activity guidelines for seniors. Once estimates of travel behavior are available, the approach is straightforward and based on relatively simple map algebra operations.

**Results:**

Compliance Potential Mapping as a tool to assess the potential contributions of walking towards physical activity is demonstrated using data from Montreal’s 2008 travel survey. The results indicate that the central parts of Montreal Island display higher potential for compliance with physical activity guidelines, but with variations according to age, income, occupation, possession of driver’s license and vehicle, and neighborhood and accessibility parameters.

**Conclusions:**

Compliance Potential Maps offer valuable information for public health and transportation planning and policy analysis.

## Background

Regular physical activity is beneficial across the lifespan. For children and youth it provides numerous health benefits and moreover has long-lasting effects [1]. At the other extreme of the life course, physical activity increases the life expectancies of older adults and decreases their risk of developing common chronic diseases [2,3]. Active travel is increasingly seen as an important source of physical activity, to the extent that it has been identified as one of the big issues in preventive medicine [4], and even called the perfect preventive medicine [5]. It is an inexpensive form of transportation that can introduce physical activity while meeting daily mobility needs [6]. Moreover walking, because of its low risk of injury, is the top recommended physical activity for seniors and is available in different settings throughout the year [7,8]. It is thus of interest to understand the factors that influence walking behavior, and its potential contributions to physical activity.

In this paper, Compliance Potential Mapping (CPM) is introduced to assess the level of physical activity associated with walking for transportation. CPM is based on the analysis of travel behavior, with due consideration to individual demographic and socio-economic attributes, the availability of mobility tools (e.g. possession of driver’s license and household vehicle), and the characteristics of the built environment. Geographical analysis of walking behavior produces maps of estimates of walking trip distance and frequency. These maps are overlaid to generate estimates of expected total walking distance, which are in turn related to physical activity.

The approach proposed is sensitive to variations in travel behavior by individual and locational attributes, and can be used to conduct very detailed analyses of walking as a source of physical activity. CPM is demonstrated for older adults using the city of Montreal as a case study.

## Methods

### Estimates of travel behavior

Generation of Compliance Potential Maps is a straightforward procedure that depends on the estimation of two elements of travel behavior, namely walking trip distance and frequency. In general terms, suppose that these estimates can be obtained by means of statistical models, as follows:

(1) $${\hat{d}}_{\mathrm{pi}}=f\left(Z_{\mathrm{pi}}\hat{\theta }\right)$$

(2) $$P\left({\hat{t}}_{\mathrm{pi}}=k\right)=g\left(X_{\mathrm{pi}}\hat{\beta }\right)$$

The ${\hat{d}}_{\mathrm{pi}}$ in Eq. (1) is the distance of a walking trip for individual *p* at location *i*, estimated as a function of a set of variables ***Z*** and with parameters ***θ***. The probability *P* in equation (2) is the number of estimated trips undertaken by individual *p* at location *i*, Further, the probability that the number of trips ${\hat{t}}_{\mathrm{pi}}$ is equal to class frequency *k* is estimated as a function of variables ***X*** and estimated parameters ***β***. It is desirable that the estimates of travel behavior reflect variations in the attributes of the individual, including age, employment status, household structure, and built environment.

There are numerous specific modeling approaches that could be adopted, some of which are briefly discussed next.

### Modeling approach

There are numerous approaches available for modeling trip distance and frequency.

Trip distances in the travel behavior literatures are usually estimated in a linear or log-linear form [9-11]. The estimation methods also vary from a simple ordinary least squares regression to more sophisticated utility-based hazard duration model. An important consideration in this context is the use of different transportation modes, since they are important in determining the trip lengths of individuals. Ignoring the propensity to use different modes can lead to endogeneity effects in the model. A simple way of accounting for variations by mode used is to introduce indicator variables for different modes e.g. [11]. A more sophisticated way of dealing with trip distances by different transportation modes is to implement a joint discrete-continuous modeling framework, which explicitly accounts for the endogeneity in the two decision processes (mode choice and distance to travel) [12,13].

The discrete part of the joint discrete-continuous model is the well-known Multinomial Logit (MNL) model and the continuous part is the continuous time hazard-based model. The MNL part assumes that an individual will choose the alternative that gives the highest utility. On the other hand, the continuous time hazard model is used to estimate the trip length for a particular trip of the individual given a specific mode of transportation. The dependent variable, trip distance, is log transformed and it thus provides a log-normal hazard model. In order to jointly estimate the discrete and continuous part of the models, the correlation among the error terms of the two models is specified. The probability that senior *p* at location *i* selects a transportation mode *m* and travel a positive distance of *d*_*pi*_ by the transportation mode can be written as:

(3) $$\begin{array}{l} \mathrm{Pr}\left(\mathrm{Distance}={\hat{d}}_{\mathrm{mpi}}\cap \mathrm{Mode}=m\right)=\mathrm{Pr}\left(\mathrm{Distance}={\hat{d}}_{\mathrm{mpi}}\cap \epsilon \leq J_{1}\left(\epsilon_{m}\right)\right)\\ =\frac{1}{\sigma_{\mathrm{mt}}{\hat{d}}_{\mathrm{mpi}}}\phi \left(\frac{\ln\left({\hat{d}}_{\mathrm{mpi}}\right)-\theta_{m}Z_{m}}{\sigma_{\mathrm{mt}}}\right)\Phi \left(\frac{J_{1}\left(\epsilon_{m}\right)-\rho_{\mathrm{mt}}J_{2}\left(\alpha_{\mathrm{mt}}\right)}{\sqrt{1-\rho_{\mathrm{mt}}^{2}}}\right) \end{array}$$

The marginal distributions of the two transformed random variables are expressed as equivalent standard normal variables, namely *J*_1_(*ϵ*_*m*_) and *J*_2_(*α*_*mt*_), and both are assumed to follow a bivariate normal distribution. The parameters are estimated under the distribution accordingly see for more details: [13].

Trip frequency can be modeled using probabilistic approaches – for instance, truncated normal, Poisson, or negative binomial models used to estimate trip frequencies. These techniques have gained popularity over the past decade [14], and are preferred over linear models because of the unrealistic output from the linear model, such as fractional or negative trip counts. Nonetheless, these probabilistic approaches do not provide a direct link to behavioral theory. Discrete choice models, on the other hand, are based on the theory of random utility and provide a more solid behavioral foundation [15]. Univariate ordinal models, such as the ordered probit or logit models, can be used to model trips by a single mode or by multiple modes pooled. A more sophisticated approach is to implement a multivariate ordered model to jointly analyze trip frequencies by each of several modes. This is preferred over estimating two or more separately univariate models, as the multivariate model account for common unobserved factors in the behavior of interest and estimates the probabilities under one formulation [16,17].

Assume that the same senior *p* at location *i* has *j* number of ordered interrelated decisions to be made simultaneously, namely the number of trips to be made by each mode of transportation *m*. Therefore, the model structure with the ordered responses can be written as:

(4) $$\begin{array}{c} t_{1\mathrm{pi}}^{*}=\beta_{1}x_{1\mathrm{pi}}+\epsilon_{1\mathrm{pi}},y_{1\mathrm{pi}}=m,\;\mathrm{if}\;\mathrm{and}\;\mathrm{only}\;\mathrm{if}\;\mu_{m,1}<t_{1\mathrm{pi}}^{*}\leq \mu_{m+1,1},\\ t_{2\mathrm{pi}}^{*}=\beta_{2}x_{2\mathrm{pi}}+\epsilon_{2\mathrm{pi}},y_{2\mathrm{pi}}=n,\;\mathrm{if}\;\mathrm{and}\;\mathrm{only}\;\mathrm{if}\;\mu_{n,2}<t_{2\mathrm{pi}}^{*}\leq \mu_{n+1,2},\\ .\\ .\\ t_{\mathrm{mpi}}^{*}=\beta_{j}x_{\mathrm{jpi}}+\epsilon_{\mathrm{jpi}},y_{\mathrm{jpi}}=o,\;\mathrm{if}\;\mathrm{and}\;\mathrm{only}\;\mathrm{if}\;\mu_{o,j}<t_{\mathrm{mpi}}^{*}\leq \mu_{o+1,j} \end{array}$$

where *y*_*1p*_, *y*_*2p*_, ……, *y*_*jp*_ are the observed number of ordered responses made by individual *p*. Depending on the *j* i.e. number of ordered responses, the model structure can take different forms – for instance, a bivariate ordered probit structure when there are two ordered responses, a trivariate ordered probit when there are three ordered responses, and so on and so forth. The set of error terms *ϵ*_*p*_ *= (ϵ*_*1p*_*, ϵ*_*2p*_*,…., ϵ*_*jp*_*)* are assumed to be distributed as multivariate normal with zero mean and *R* correlation matrix. Nonzero elements in the correlation matrix represent the correlations of common unobserved factors in the ordered decisions [18].

Regardless of the approach selected, the use of spatial models is desirable for the ability to introduce spatially-varying parameters in mobility models. Available techniques include the spatial expansion method [9,10,19,20] or multi-level models [11,21,22].

### Compliance potential maps

**Figure 1:**
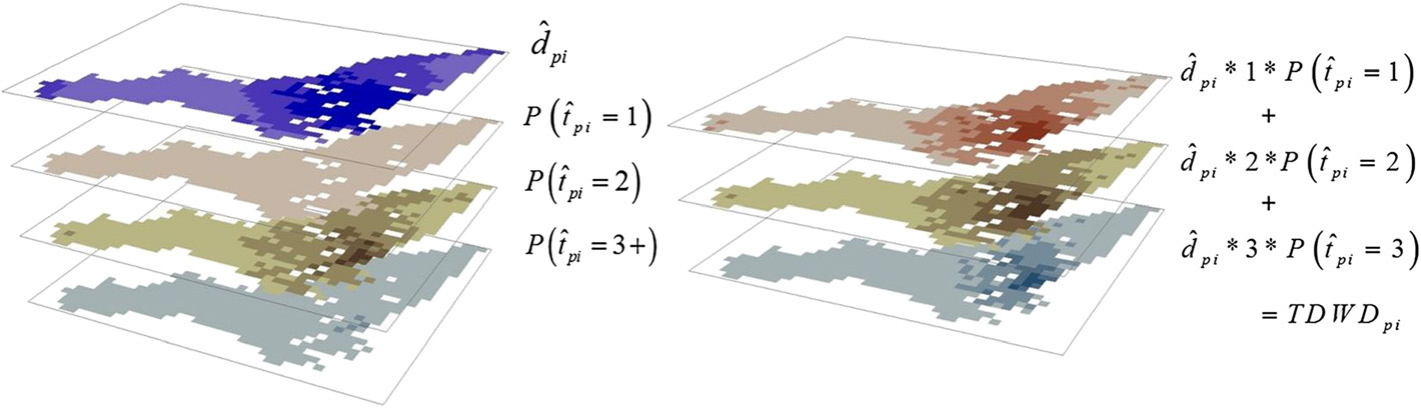
Overlay of map layers and calculation of trip distance for trip frequencies.

Given estimates of travel behavior, Compliance Potential Maps can be generated by means of map algebra operations, as shown in Figure 1. However, as we focus on walking because of its contribution towards physical activity among the elderly age cohorts whereas other two modes do not and therefore estimation of those modes were not used in the subsequent analysis in this study. The top layer on the left consists of estimates of walking trip distance. Additional layers are estimates of the probability that walking trip frequency is *k* (e.g. 1, 2, and 3+). The expected walking distance corresponding to each trip class is obtained multiplying the estimated trip distance times *k*, times the probability that the frequency of walking trips is *k*. These map algebra result in a set of *K* layers as shown on the right side of Figure 1. Estimates of total daily walking distance (TDWD) are obtained overlaying all expected trip distance layers, so that for each geographical sub-unit *i* (e.g. Dissemination Area, Census Tract, Traffic Analysis Zone, Zip Code etc.) in a map TDWD is:

(5) $$\mathrm{TDW}D_{\mathrm{pi}}=\;\sum_{k=1}^{K}{\hat{d}}_{\mathrm{pi}}*\;k\;*\;P\left({\hat{t}}_{\mathrm{pi}}=k\right)$$

The potential for compliance with physical activity guidelines is evaluated as follows. First, it is assumed that the daily behavior is repeated over *w* days every week (e.g. five days a week). Total weekly distance is estimated multiplying *TDWD* by *w*. Then, using a suitable value for walking speed *s*, the weekly distance is converted to total weekly walking minutes. For instance, Montufar et al. [23] proposed an average walking speed of 68.4 m/min for seniors. Using the selected value for speed, the weekly walking distance is then converted into weekly walking minutes:

(6) $$\mathrm{WW}M_{\mathrm{pi}}=\;\left(\mathrm{TDW}D_{\mathrm{pi}}*w\right)/s$$

According to the New Canadian Physical Activity Guidelines, weekly recommended minimum physical activity requirements for the seniors are 2.5 hours or 150 minutes [24]. Considering an average walking speed of 68.4 m/min for seniors [23], 150 min of physical activity is equivalent to 10.26 km of walking per week. Finally in compliance with the guidelines, weekly walking minutes are converted into the percentage of physical activity recommended in relevant guidelines, such as the New Canadian Physical Activity Guidelines [24,25].

### Materials

To illustrate the concept of Compliance Potential Mapping, the case of older adults in Montreal Island, Canada, is considered. Montreal Island is the most populous as well as developed part of the Montreal Census Metropolitan Area. The Island, like many North American cities, has been facing a tremendous population growth as well as a rapid growth in the senior age cohorts and this will continue to increase until 2061. According to the statistics of the *Institut de la Statistique du Québec* (ISQ), some census subdivisions in the Island exceed over 60% of its population in the seniors age cohorts i.e. 65 years or older more details about the context can be found in: [13].

Data for the analysis are drawn from the 2008 Montreal Household Travel Survey database. The database is an outcome of Origin–destination (OD) survey which was firstly started in 1970 and the 2008 database is ninth in the series. September 3rd to December 19th of 2008 was time frame when the 2008 OD survey was conducted. A Computer Aided Telephone Interview (CATI) instrument was used in contracting the participants and data were only recorded when a participant performed out-of-home activities on the survey day. Various trip identifiers (e.g. address, nearest intersections, trip generators) were used to geocode the trip origin and destination in the database, to produce high quality location references. Given the scale of the survey, it is impossible to implement other direct approaches, such as observation using GPS traces. It is important to note as well that this is a secondary data source and therefore does not require ethics approval.

Senior trip-makers and a transitional age cohort, 55–64 years, were taken for this analysis. The dataset was cleaned in several ways. For instance, there were only a few student seniors or seniors who are lone parents of young children. These records were removed because of their extremely low variability. Moreover, only home-based trips (i.e. either origin or destination of a trip is home of the trip maker) with a distance greater than zero were included to account for the location of each trip’s origin or destination (i.e. use of trend surface) in estimating the two elements of walking behavior. In terms of transportation modes, the three most popular among seniors (car, transit, and walk) were included in the final dataset. The trips made by car include both as a driver and as a passenger while removing cycling being a rare transportation mode among the groups. Finally, a total of 31,631 trips made by 13,127 individual seniors aged 55 or over were available for analysis. The distribution of the total trips is walking 17.35%, transit 19.44%, and car 63.21%. Average trip distance for seniors across the three modes included in this study is 5.33 km, whereas average walking trip distance is 0.74 km, with a standard deviation of 8.44 and 1.06 km, respectively. Average trip frequency for all modes is 2.41 and for walking 2.16. In addition to mobility information, built environment variables (including street density, intersection density, and land use mix) were created from the 2009 DMTI spatial [26] database. It is noteworthy to mention that although the Montreal Household Travel Survey targets a sample size of 5% of total population across the Montreal Census Metropolitan Area, the DMTI database for built environment was not so rich outside of Montreal Island and therefore we confined our analysis within the Island only. The unit of aggregation for the built environment variables is the Dissemination Area (DA), for compatibility with the smallest publicly available Census geography in Canada. Finally, six accessibility-related variables were also calculated to include in this study, namely the number of activity locations within a 400 m buffer of the place of residence, and network distance to nearest essential facilities (pharmacy, health facility, bank, grocery, and library). Environics Analytics a Toronto-based market firm and infoCanada produced a geocoded business location database that was used to calculate the accessibility variables.

In-depth discussions regarding variable selection can be found in Moniruzzaman et al. [13], Morency et al. [10] and Roorda et al. [19]. The variables selected were tested for collinearity, and it was found that the correlations did not exceed *R* = 0.5.

## Results and discussion

The results of the models are shown in Tables 1 and 2. Some highlights of the models are discussed in this section.

**Table 1:** Estimates for the continuous part (trip distance) of the joint discrete-continuous model, Montreal 2008

	**Walking**		**Car**		**Transit**	
	**Coefficient**	***p*-value**	**Coefficient**	***p*-value**	**Coefficient**	***p*-value**
**Constant**	2.2718	0.000	Reference		−3.1657	0.000
**Personal characteristics**						
Age: Younger senior	Reference		Reference		Reference	
Age: Senior	−0.1261	0.001	−0.0639	0.001	−0.1022	0.006
Age: Elder senior	−0.2215	0.000	−0.1484	0.000	−0.2983	0.000
Gender	0.0284	0.351	−0.1157	0.000	−0.0609	0.035
Household: Single	Reference		Reference		Reference	
Household: Couple	0.0767	0.031	−0.0457	0.032	−0.0095	0.781
Household: Other	0.1073	0.025	−0.0865	0.001	0.0459	0.269
Occupation: Full-time	−0.2132	0.000	0.3576	0.000	0.4770	0.000
Occupation: Part-time	−0.0892	0.145	0.1811	0.000	0.3377	0.000
Occupation: Retired	Reference		Reference		Reference	
Occupation: At-home	−0.0021	0.978	−0.1248	0.011	−0.0572	0.448
Income: <20 k	Reference		Reference		Reference	
Income: 20–40 k	−0.0265	0.558	−0.0519	0.129	0.0294	0.482
Income: 40–60 k	0.0525	0.363	0.0763	0.031	0.0382	0.463
Income: 60–80 k	0.0681	0.327	0.0200	0.605	0.0627	0.341
Income: 80–100 k	0.2877	0.000	0.1718	0.000	−0.0355	0.673
Income: g100k	0.3490	0.000	0.0561	0.161	−0.1444	0.056
Income: RF/DK	0.0949	0.027	−0.0029	0.928	0.0048	0.908
**Mobility tools**						
Driver licence	0.0769	0.041	0.1173	0.000	−0.0200	0.528
Vehicle own: 0	Reference		Reference		Reference	
Vehicle own: 1	−0.3596	0.000	0.4320	0.000	−0.2659	0.000
Vehicle own: 2	−0.5863	0.000	0.6270	0.000	−0.4162	0.000
Vehicle own: 3+	−0.7427	0.000	0.5734	0.000	−0.2216	0.072
**Neighborhood characteristics**						
Population density: Low	Reference		Reference		Reference	
Population density: Med	−0.0345	0.573	−0.0545	0.022	0.1083	0.057
Population density: High	−0.0541	0.389	−0.0876	0.001	0.072	0.210
Job density: Low	Reference		Reference		Reference	
Job density: Medium	0.0927	0.008	−0.0716	0.000	0.2022	0.000
Job density: High	0.1607	0.001	−0.0040	0.877	0.7646	0.000
Street density	0.0109	0.008	−0.0080	0.001	−0.0001	0.989
Intersection den	−0.0009	0.022	0.0007	0.010	−0.0001	0.868
BSF to DA	−0.0966	0.550	−0.2363	0.018	−0.4847	0.004
Land use mix	−0.0159	0.802	−0.0336	0.343	0.0074	0.913
**Accessibility**						
Activity locations (400 m)	−0.7184	0.002	0.0592	0.732	0.0939	0.720
Nearest pharmacy	0.2214	0.000	0.0291	0.163	−0.1171	0.022
Nearest health facility	0.1308	0.150	−0.0685	0.032	−0.0121	0.889
Nearest bank	−0.1958	0.000	0.1343	0.000	0.0784	0.026
Nearest grocery	0.5091	0.000	−0.0208	0.514	−0.0187	0.828
Nearest library	0.0371	0.018	−0.0054	0.443	0.0075	0.610
**Trend surface (quadratic)**						
Latitude	−1.6621	0.000	0.0405	0.513	1.5200	0.000
Longitude	−0.6857	0.006	0.2362	0.002	0.6818	0.006
Latitude*Longitude	0.0043	0.956	−0.0885	0.008	−0.0884	0.255
Latitude squared	0.2301	0.000	0.0283	0.139	−0.1932	0.002
Longitude squared	0.3994	0.000	−0.0116	0.628	−0.3064	0.000
Distance to CBD (km)	−1.2353	0.000	0.2750	0.000	1.4113	0.000
**Summary statistics**						
Number of observations = 31,631			Correlation between discrete and continuous models = −0.472		Sigma = 1.084	
Log Likelihood (Constant only model) = −77,468.43			Log Likelihood (Full model) = −65,771.60			
McFadden's adjusted-*ρ*^2^ = 0.1485			Likelihood ratio test = 23,393.66 (*p* < 0.0001)			

Note: gray cells indicate *p* > 0.10.

**Table 2:** Estimates of the multivariate ordered probit models for home-based trips by mode, Montreal 2008

	**Walking**		**Car**		**Transit**	
	**Coefficient**	***p*-value**	**Coefficient**	***p*-value**	**Coefficient**	***p*-value**
**Threshold parameters**						
Threshold 1 (trip = 0)	−3.5794	0.000	0.9163	0.000	−0.7470	0.000
Threshold 2 (trip = 1)	−3.4745	0.000	1.0759	0.000	−0.9343	0.000
Threshold 2 (trip = 2)	−2.2748	0.000	3.0781	0.000	2.7782	0.000
**Personal characteristics**						
Age: Younger senior	Reference		Reference		Reference	
Age: Senior	−0.0900	0.007	0.0864	0.001	−0.0611	0.070
Age: Elder senior	−0.1278	0.001	0.1375	0.000	−0.2368	0.000
Gender	-	-	−0.0748	0.001	0.0975	0.000
Household: Single	Reference		Reference		Reference	
Household: Couple	−0.0730	0.022	−0.1030	0.000	0.0527	0.100
Household: Other	−0.1182	0.005	−0.2213	0.000	0.2155	0.000
Occupation: Full-time	−0.3630	0.000	−0.0725	0.009	0.2961	0.000
Occupation: Part-time	−0.1415	0.007	-	-	0.2341	0.000
Occupation: Retired	Reference		Reference		Reference	
Occupation: At-home	-	-	-	-	-	-
Income: <20 k	Reference		Reference		Reference	
Income: 20–40 k	-	-	-	-	0.1242	0.000
Income: 40–60 k	−0.0763	0.058	0.1444	0.000	-	-
Income: 60–80 k	-	-	0.1729	0.000	0.0918	0.061
Income: 80–100 k	0.1077	0.088	0.0899	0.051	-	-
Income: g100k	0.1099	0.044	0.2007	0.000	−0.1051	0.050
Income: RF/DK	-	-	-	-	-	-
**Mobility tools**						
Driver licence	−0.2120	0.000	0.4791	0.000	−0.3722	0.000
Vehicle own: 0	Reference		Reference		Reference	
Vehicle own: 1	−0.3919	0.000	1.4216	0.000	−1.0726	0.000
Vehicle own: 2	−0.6307	0.000	1.6987	0.000	−1.4726	0.000
Vehicle own: 3+	−0.6145	0.000	1.6615	0.000	−1.5867	0.000
**Neighborhood characteristics**						
Population density: Low	Reference		Reference		Reference	
Population density: Med	-	-	-	-	-	-
Population density: High	-	-	−0.0456	0.046	-	-
Job density: Low	Reference		Reference		Reference	
Job density: Medium	−0.1199	0.000	−0.0569	0.022	0.2988	0.000
Job density: High	−0.3352	0.000	−0.5265	0.000	0.9346	0.000
Street density	0.0039	0.100	−0.0049	0.016	0.0096	0.004
Intersection den	-	-	-	-	−0.0009	0.009
BSF to DA	0.2426	0.091	−0.3610	0.002	-	-
Land use mix	-	-	-	-	-	-
**Accessibility**						
Activity locations (400 m)	0.1150	0.000	−0.07574	0.000	−0.1078	0.000
Nearest pharmacy	-	-	-	-	−0.0860	0.031
Nearest health facility	-	-	-	-	−0.1703	0.009
Nearest bank	−0.1425	0.000	-	-	0.0596	0.040
Nearest grocery	-	-	0.0679	0.072	-	-
Nearest library	-	-	-	-	-	-
**Trend surface (quadratic)**						
Latitude	−1.6413	0.000	0.0885	0.014	.5818	0.000
Longitude	−0.6904	0.000	-	-	-	-
Latitude*Longitude	-	-	-	-	-	-
Latitude squared	0.2335	0.000	-	-	−0.1214	0.000
Longitude squared	0.3873	0.000	−0.0318	0.001	-	-
Distance to CBD (km)	−1.1640	0.000	0.1558	0.000	-	-
**Summary statistics**						
Number of observations = 13,127	LL (Null model) = −26223.78				LL (Full model) = −22408.69	
*Correlation*	walking and car = −0.474		walking and transit = −0.231		car and transit = −0.656	
McFadden's adjusted-*ρ*^2^ = 0.1484					Likelihood ratio = 7630.18	

### Mode choice and trip distance

A joint discrete-continuous model was estimated for mode choice and trip distance by mode.

The results of the analysis are in line with previous research on mobility by older adults. Firstly, it is found that trips become shorter with age, irrespective of mode of transportation. Compared to retired seniors, all working seniors tend to make shorter walking trips, and longer car and transit trips, as expected given time budget constraints. Higher income seniors (>$80 k) make longer walking trips when compared to lower income seniors (<$20 k). Possession of driver’s license and number of vehicles owned by the senior household decrease the walking and transit trip distances among seniors and increase the car trip distance which was also found in the past studies [11]. Employment and street density contribute positively towards longer walking trips. More activity locations around the senior’s place of residence decreases the distance of walking trips, but do not influence trip distance by car and transit. The distance to nearest pharmacy, grocery store, and library increases the distance of walking trips, whereas distance to the nearest bank hand decreases it.

### Trip frequency

A multivariate ordered probit model was obtained for trip-making frequency of walking, car, and transit.

The results indicate that with increasing age seniors become more likely to make fewer walking trips. Compared to single seniors, seniors who live with a couple are less likely to make more trips, perhaps due to division of responsibilities [20]. Full-time and part-time seniors make fewer walking trips. Higher income seniors tend to make fewer walking trips than lower income seniors. Access to the mobility tools tends to increase the frequency of car trips.

Seniors in the medium, and high job density areas are less likely to make more walking, and car trips and more likely to make trips by transit. Street density and square footage to building ratio are also significant in determining the propensity of higher walking trips and the effect of these variables are negative for car trips. Land use mix is not however significant for any of the trip frequency models. Number of activity locations within a 400 m buffer around the senior’s residence is positively associated with the walking trip-making propensity. Similar findings were found in past elderly studies on walking [27,28].

### Geographic variations in trip distance and frequency

**Figure 2:**
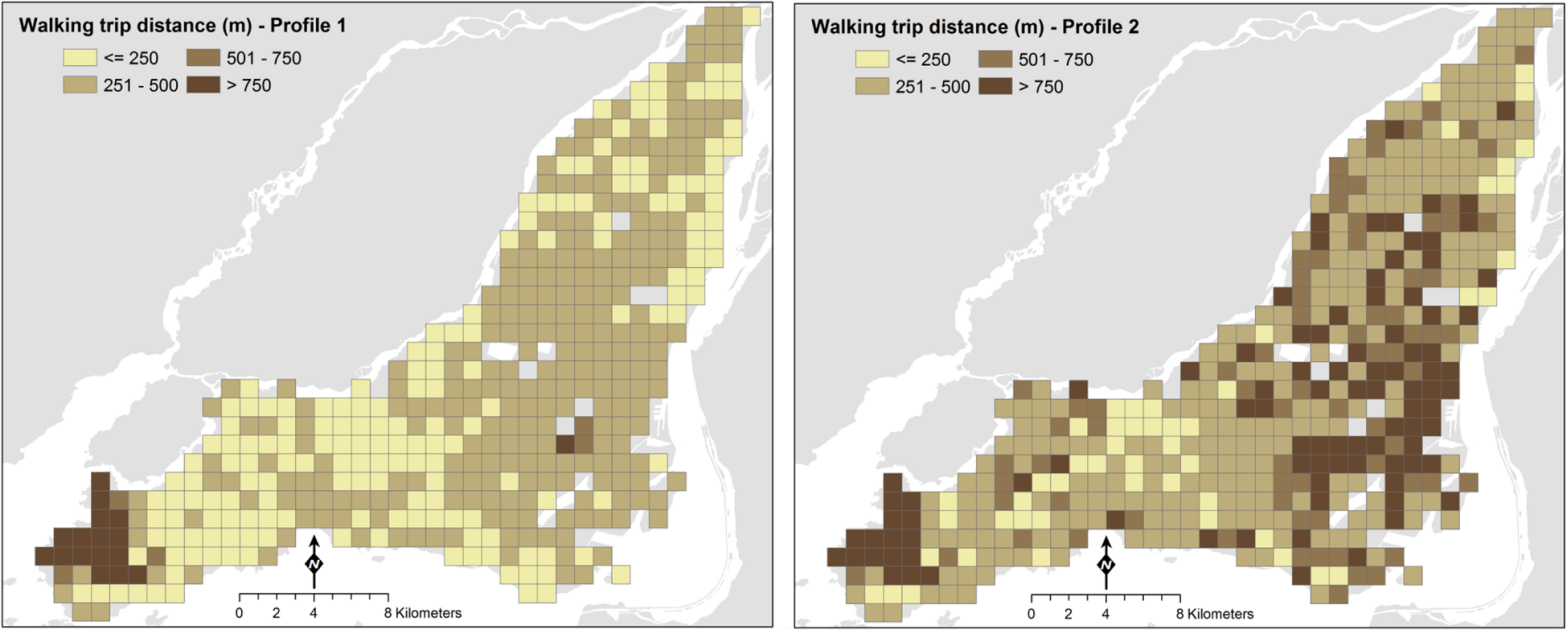
Geographic variations in walking trip distances, Montreal 2008.

Geographic variations in mobility behavior over space, even within an otherwise uniform cohort, are captured by means of a trend surface that combines distance from central business district (CBD) and a quadratic function of the coordinates of the senior’s home location. One of the advantages of including this trend surface is that it captures the geographic variations of the behavior of interest assuming that the components of the trend surface are statistically significant. In other words, use the geographic attributes as independent variables into the models might capture the geographic variations in the dependent variable when their effects are statistically significant. Another advantage of this approach is that it allows complete visualization of the variations, if any, in the dependent variable. For instance, it is possible to show the geographic variations of the trip distance and frequency in particular for this analysis. To do that adequate number of square grids (250 m × 250 m) are superimposed on the study area of interest and then the coordinates of the grid centroid are used to estimate the variations of the behaviors. This grid is used for visualization purposes only, and the size of the cells has no impact whatsoever on the preceding analysis.As an example, consider two different senior profiles selected to contrast the variations in behaviors. The profiles are as follows. Profile 1 is a retired male or female (effect is same as gender is non-significant in any of the models), aged between 65 and 74 years, and with an income less than 20 thousand dollars. The profile also assumes that the senior lives with a couple, and does not own a driver license or an automobile. Profile 2 is a retired male or female, aged between 65 and 74 years, with an income of 80–100 thousand dollars. The profile assumes that the individual owns a driver license and an automobile. Estimated walking trip distances for each of these profiles across Montreal are shown in Figure 2.

**Figure 3:**
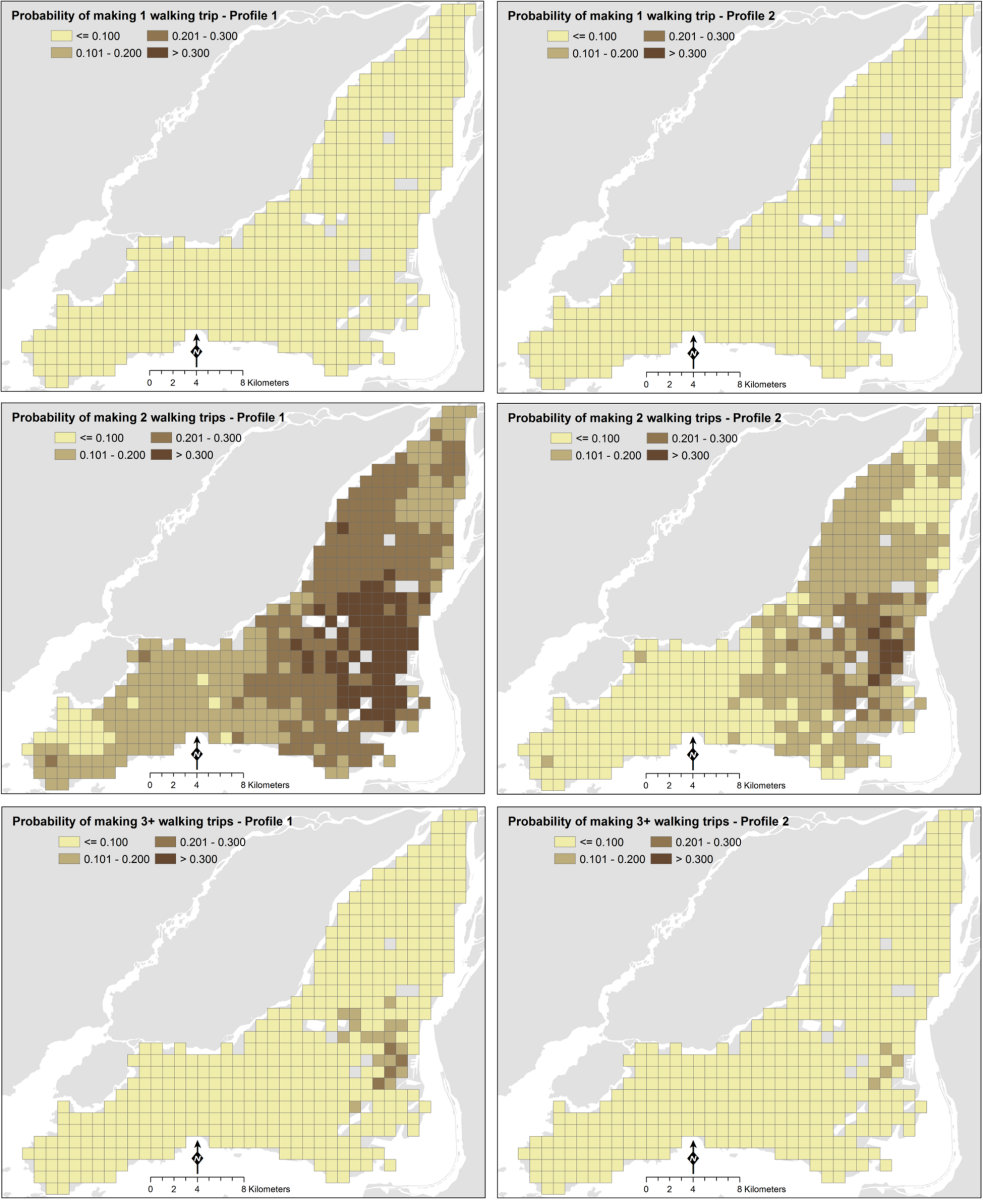
Geographic variations in probabilities of walking trip frequency, Montreal 2008.

The probabilities of undertaking 1, 2, and 3+ walking trips were estimated for the same senior profiles based on the multivariate ordered probit model. Three or more walking trips were combined into a same trip frequency class as very few seniors in the dataset undertook four or more walking trips. The results are shown in Figure 3. The maps in Figures 1 and 2 are the basis for estimating *TDWD* and *WWM*.

### Compliance potential maps

**Figure 4:**
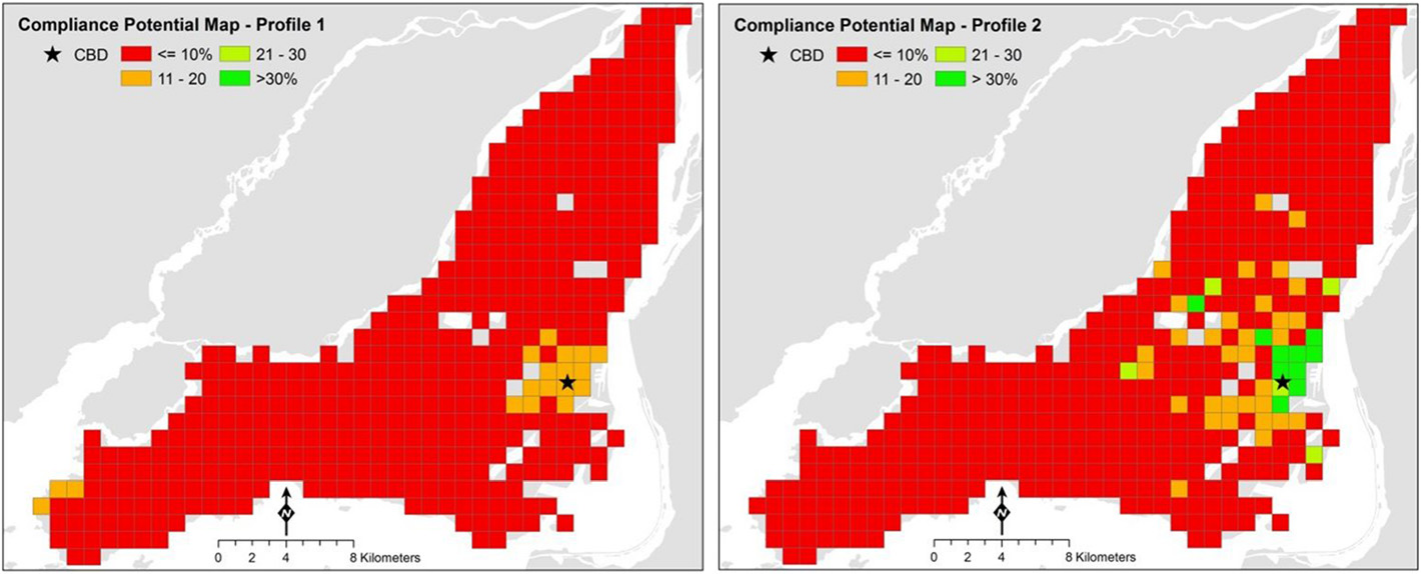
Compliance Potential Maps for two selected senior profiles, Montreal 2008.

Trip distance and frequency probability maps are used to calculate *TDWD* as per Eq. (5) and *WWM* is estimated using Eq. (6). Finally the estimated *WWM* is converted into the percentage of minimum physical activity compliance prescribed in New Canadian Physical Activity Guidelines [24,25].Compliance Potential Maps are shown in Figure 4 for the two individual profiles. The variable underlying each grid cell indicates the estimated contribution towards physical activity guidelines (in percentage) that seniors characterized by Profiles 1 and 2 can derive from walking for transportation. The maps are produced assuming that mobility behavior is repeated for five days a week.

The differences between Profiles 1 and 2 lie in the income levels and access to mobility tools (driver’s license and automobile). This difference translates in relatively large variations in the potential for compliance. It can also be observed in both compliance maps that seniors in the central part of the Montreal Island have higher potential for compliance, but that this effect tends to decrease with distance from the CBD. Since compliance estimates are obtained from the models of travel behavior it is possible to assess the factors that influence compliance. In reference to Tables 1 and 2, it can be seen that the concentration of activity locations in the CBD area tends to increase the propensity for walking trips, but decrease the distance of trips. Employment density also tends to be higher in that region, which increases trip distance but reduces the propensity for trips. The opposite is true in more suburban regions.

**Figure 5:**
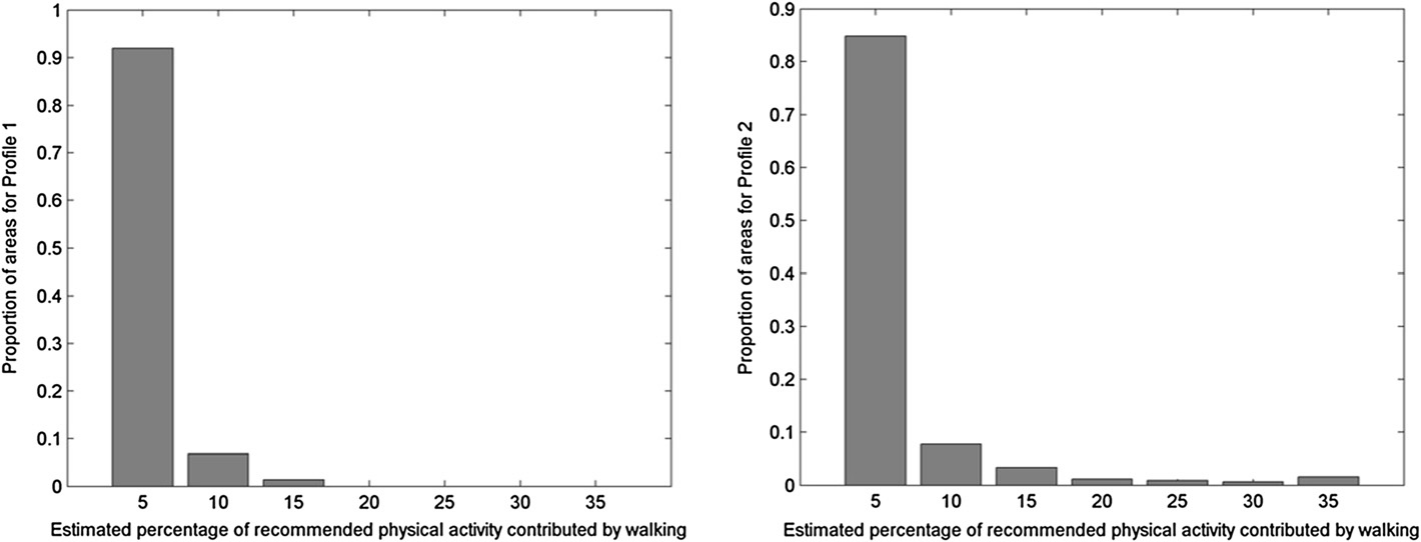
Distribution of areas according to compliance potential for Profile 1 (left panel) and Profile 2 (right panel).

Figure 5 shows the distribution of areas according to compliance potential. It can be seen there that the maximum potential for compliance based on walking for Profile 1 is approximately 15%. In contrast, there is a relatively large area (approximately 15 km^2^) in the surroundings of downtown Montreal where Profile 2 potentially can achieve in excess of 20% of recommended physical activity by walking, and in some locations greater than 30%. It is can be appreciated that more affluent seniors with access to personal automobile have a greater potential for compliance from walking alone, than the relatively poor seniors without access to a personal automobile represented by Profile 2. While Profile 1 seniors have a greater propensity for walking trips, their trips tend to be substantially shorter. In this case, fewer but longer trips are the key to higher compliance. Similarly, it can be appreciated that for seniors in suburban areas walking for transportation contributes only marginally towards physical activity guidelines. Given the limited benefits provided by active travel, encouraging physical activity during leisure time for suburban seniors would appear to be of greater urgency.

## Conclusion

In this paper the concept of Compliance Potential Mapping was introduced and the approach was demonstrated by means of case study of seniors in Montreal. Models of walking behavior show that the walking trip distance and frequency of seniors are determined by individual attributes, mobility tools, as well as the accessibility and built environment. The use of a travel diary data with large sample size (i.e. number of trips) significantly validates the model results. Spatial analysis reveals significant geographical variations on walking behavior. Mapping these variations has potentially useful policy applications. Compliance Potential Maps for two individual profiles help to identify the potential contributions of walking for transport to physical activity at various locations within Montreal. It is important to note that maps displayed in the paper are illustrative of the applicability of the approach, and the models allow a great degree of flexibility in defining the individual profile for detailed analysis. Travel surveys are collected in many major urban regions, and provide a rich source of information that can be used to generate Compliance Potential Maps. On the other hand, given the characteristics of the data (1 day travel diary), some assumptions are required. First, it was assumed that the daily behavior is repeated over five days a week. This gives a conservative estimate by excluding weekend days. Additional study with week-long travel diary data is suggested as a matter for future research. Secondly, trips were not directly observed, for instance using GPS traces, but reported by participants in the survey. Therefore, some assumptions had to be made regarding constant displacement speed (i.e. 68.4 m/min). High quality industry standards were used in the collection of the data, but there is always the potential for some recall bias. Use of GPS, although promising as a way of collecting data about trips in a more direct fashion, poses important challenges, from the logistics of delivering equipment to respondents, to the quality of data in urban environments. GPS-based surveys tend to be small. The travel survey used in this paper, on the other hand, includes tens of thousands of trips. Extent of coverage in this case is preferred to (potentially) higher levels of accuracy in data recording, and recall bias may become less of an issue given the large sample size.

The concept of Compliance Potential Mapping is very general, and provides a systematic way to assess health and transportation policies. These maps can be used in a number of ways. First, the maps help to identify areas where compliance with physical activity guidelines is potentially low. Secondly, the models could be used to assess changes in any of the variables to assess the anticipated impact of changes in population or built density, street and intersection densities, or the availability of activity locations and services. Urban features that contribute towards physical activity guidelines can be investigated in detail, and their differential impact on various individual profiles and locations evaluated. Thirdly, the tool could be used to target neighborhoods for policy interventions, in order to help to use resources efficiently. Alternatively, these maps could be used to guide purposive data collection for qualitative research. The flexibility and potential of the suggested applications should make Compliance Potential Mapping an interesting tool for public health professionals, urban and transport planners, and policy makers.

## Competing interests

The authors declare that they have no competing interests. This research was supported financially by the Canadian Institutes for Health Research.

## Authors’ contributions

The concept was developed by AP and MM, CM was responsible for data processing. Statistical analysis was performed by MM under the supervision of AP. All authors contributed to writing the manuscript. All authors read and approved the final manuscript.

## Pre-publication history

The pre-publication history for this paper can be accessed here:

http://www.biomedcentral.com/1471-2458/14/511/prepub
